# Shift in Phenotypic Characteristics of Enterotoxigenic *Escherichia coli* (ETEC) Isolated from Diarrheal Patients in Bangladesh

**DOI:** 10.1371/journal.pntd.0003031

**Published:** 2014-07-17

**Authors:** Yasmin Ara Begum, Nabilah Ibnat Baby, Abu S. G. Faruque, Nusrat Jahan, Alejandro Cravioto, Ann-Mari Svennerholm, Firdausi Qadri

**Affiliations:** 1 International Centre for Diarrheal Disease Research, Bangladesh (icddr,b), Dhaka, Bangladesh; 2 International Vaccine Institute, Seoul, Korea; 3 Institute of Biomedicine, Department of Microbiology and Immunology, University of Gothenburg, Gothenburg, Sweden; Oxford University Clinical Research Unit, Viet Nam

## Abstract

**Background:**

Enterotoxigenic *Escherichia coli* (ETEC) is one of the most common causes of bacterial diarrhea. Over the last decade, from 1996 to 2012, changes in the virulence antigen properties of ETEC such as heat labile (LT) and heat stable (ST) toxins, colonization factors (CFs), and ‘O’-serogroups have been observed. The aim of this prospective study was to compare changes in antigenic profiles of ETEC strains isolated from a 2% surveillance system at the icddr,b hospital in Dhaka, Bangladesh between 2007–2012 and an earlier time period of 1996–1998 conducted at the same surveillance site.

**Methodology:**

In the surveillance system every 50^th^ patient attending the hospital was screened for major enteric pathogens including ETEC, *Vibrio cholerae*, *Shigella* spp. and *Salmonella* spp. from January 2007 to December 2012.

**Principal Findings:**

Of the 15,152 diarrheal specimens tested between 2007–2012, the overall rate of ETEC isolation was 11%; of these, 43% were LT/ST, 27% LT and 30% ST positive. Isolation rate of ST-ETEC (p<0.009) and LT/ST ETEC (p<0.011) during 2007–2012 period differed significantly compared to those seen between 1996–1998. In comparison to the 1996–1998 period, difference in CF profile of ETEC isolates during 2007–2012 was observed particularly for strains expressing CS7 (12.4%), CS14 (9.5%) and CS17 (10.0%). The predominant CF types were CS5+CS6, CFA/I, CS7, CS17, CS1+CS3, CS6 and CS14. The most common serogroups among the CF positive ETEC isolates were O115, O114, O6, O25 and O8. A strong association was found between CFs and ‘O’ serogroups i.e. between CS5+CS6 and (O115 and O126); CS7 and (O114), CFA/I and (O78 and O126), CS17 and (O8 and O167) and CS1/CS2+CS3 and (O6).

**Conclusion:**

The analyses show a shift in prevalence of antigenic types of ETEC over the study period; the information is important in designing effective ETEC vaccines with broad protective coverage.

## Author Summary

Diarrheal diseases constitute a major health problem in Bangladesh, where *Vibrio cholerae* and enterotoxigenic *Escherichia coli* (ETEC) are two most important causes of bacterial diarrhea. Prevention through vaccination is helpful to reduce the incidence and severity of diarrheal disease due to ETEC, particularly among children in low-resource settings. In this context, we collected stool and/or rectal swab (RS) specimens from patients with diarrhea between 2007 to 2012 under the 2% systematic routine surveillance system at the icddr,b hospital in Dhaka, Bangladesh and screened for ETEC infection. We tested the specimens for two major virulence factors of ETEC: toxins and colonization factors. In this research article, we have focused on changes in toxin as well as colonization factor profiles of ETEC strains isolated from diarrheal patients seeking care at the icddr,b hospital between 2007–2012 and an earlier time period of 1996–1998. We concluded that, such shift in antigenic profile of ETEC over the study period is important in designing effective ETEC vaccines with broad protective coverage.

## Introduction

Enterotoxigenic *Escherichia coli* (ETEC) is one of the leading causes of bacterial diarrhea in children and adults in developing countries. ETEC is a multivalent pathogen and have been sub-classified into different virulence types based on production of different enterotoxins, colonization factors (CFs) and the ‘O’ antigen group of the lipopolysaccharide. At least 160 ‘O’ serogroups have been found among ETEC strains isolated from humans, although a more restricted number of serogroups are detected among strains isolated from patients requiring medical intervention.

Moreover, the ETEC phenotypic heterogeneity is also illustrated by different enterotoxin profiles and expression of different CFs as well as additional putative adhesion factors [1]–[4]. ETEC strains express one or both of two enterotoxins, the heat labile toxin (LT) and the heat stable toxin (STh, STp variants) [5] and more than 25 different CFs including CFA/I and CS1–CS22 [2], [6], [7]. In this analyses, we have compared changes in antigenic profiles of ETEC strains isolated from a 2% surveillance system of all stool specimens from patients seeking care for diarrhea at the icddr,b hospital in Dhaka, Bangladesh between 2007–2012 compared to that seen during an earlier time period between 1996–1998 under the same surveillance system.

## Methods

### Study Site

This 2% systematic routine surveillance system was carried out between 2007 to 2012 in patients (n = 15,152) attending the diarrheal hospital at the icddr,b, Dhaka, Bangladesh. All patients were assessed for clinical conditions, including degree of dehydration according to WHO guidelines. The clinical criteria for admission were moderate to severe diarrhea requiring hospitalization.

### Ethics Statement

The 2% surveillance system was a routine ongoing activity of the icddr,b Dhaka Hospital which has been approved by the Research Review Committee (RRC) and Ethical Review Committee (ERC) of icddr,b. Since most of the patients were illiterate, informed oral consent was obtained from the caregivers or guardians on behalf of the patients for collecting stool specimens only, following the hospital policy. The information was stored in the hospital database and used for conducting research. The verbal consent was documented by keeping a check mark in the questionnaire which was again shown to the patient or the guardians. At the same time, patients or the guardians were assured about the non-disclosure of information collected from them, and were also informed about the use of data for analysis and using the results for improving patient care activities as well as publication without disclosing the name or identity of the participants. ERC was satisfied with the voluntary participation, maintenance of the rights of the participants and confidential handling of personal information by the hospital audit committee and approved this consent procedure.

### Microbiological Evaluation

Under the 2% systematic routine surveillance system stool and/or rectal swab (RS) specimens were collected from every 50^th^ patient attending the icddr,b hospital and screened for major enteric pathogens including ETEC, *Vibrio cholerae*, *Shigella* spp. and *Salmonella* spp. by standard microbiological and biochemical methods [8], [9]. For the detection of ETEC, each stool/rectal swab specimens were plated on MacConkey agar and the plates incubated overnight at 37°C. Six lactose fermenting, individual colonies from each stool or rectal swab were tested by multiplex PCR and ganglioside GM_1_-Enzyme- Linked Immuno Sorbent Assay (ELISA) for the presence of LT and ST of ETEC. Multiplex PCR was used to detect both variants of ST toxin, STh and STp.

### Multiplex PCR for Detection of ETEC Toxin

Multiplex PCR for enterotoxins was performed as previously described [10], [11]. Single band gene products of the correct sizes for LT, STh and STp toxin genes were obtained in 25.5 µL PCR reactions containing 1 Unit Taq polymerase (Takara), 10× PCR reaction buffer containing 25 mM MgCl_2_ (Sigma Aldrich, St. Louis, MO), 2.5 mM dNTPs (Roche, Mannheim, Germany) and 4 pmol of each primer.

### Multiplex PCR for Detection of ETEC CFs

In order to simplify and accelerate the detection of six CFs- CS13, CS15, CS18, CS17/CS19, CS20 and CS22 specific primers designed to target corresponding CF genes. These were assembled into two panels. In panel I, CS15 (*nfaA*), CS19 (*csdB*), CS20 (*csnA*) genes and in panel II, CS13 (*cshE*), CS18 (*fotG*), and CS22 (*cseA*) genes were amplified, respectively. In each panel, single band gene products of the correct sizes for CS15, CS19, CS20 as well as CS13, CS18 and CS22 genes were obtained in 25.0 µL PCR reactions containing 5 U/µL Taq polymerase (Takara), 10× PCR reaction buffer containing 25 mM MgCl_2_ (Sigma Aldrich, St. Louis, MO), 2.5 mM dNTPs (Roche, Mannheim, Germany) and 10 pmol of each primer. The PCRs were amplified by an initial denaturation at 94°C (1 min), followed by 35 cycles of amplification (94°C for 30 s, 52°C for 30 s, and 72°C for 1 min), and finally, 5 min at 72°C. Amplified products were analyzed by agarose gel electrophoresis on 3.0% agarose gel [10], [12].

### Phenotypic Detection of Toxin Profile and CFs

The detection of LT and ST was carried out by previously described GM_1_-ELISA method [8], [11]. Six lactose fermenting *E. coli* colonies (half of each) from MacConkey agar plates were inoculated on GM_1_-coated micro titer plates containing Luria Bertani broth for 18 h. The culture supernatant was tested for ST using an inhibition ELISA procedure [13] and for LT by using a LT specific monoclonal antibody [11], [14]. For ETEC positive samples, the remaining half of each colony was plated onto colonization factor antigen (CFA) agar with and without bile salts and was tested for the expression of CFA/I, CS1, CS2, CS3, CS4, CS5, CS6, CS7, CS8, CS12, CS14 and CS17 using 13 CF specific monoclonal antibodies by dot-blot immunoassay [8], [11]. The isolates were also cultured on Trypticase Soy Agar (TSA) plates and tested for CS21 only [15].

### ‘O’-Serogrouping

ETEC isolates were cultured on blood agar plates containing 5% sheep blood at 37°C. The ‘O’-antigenic serogrouping was carried out following standard method using eight commercially available polyvalent and 43 monovalent antisera (Denka Seiken) [16].

### Statistical Analysis

Comparisons were carried out where necessary using SigmaStat statistical software (Jandel scientific, San Rafael, Calif. USA). R version 2.14.1 for Chi-square test was used for significance analysis. However, missing data were very minor (<1%), thus excluded from the analysis.

## Results

### Prevalence of ETEC with Different Toxin Profiles

During 2007–2012 diarrheal specimens (n = 15,152) from patients under the 2% surveillance system were collected and analyzed for the presence of ETEC. The results were compared with those obtained for specimens collected between 1996–1998 (n = 4,662) under the same study. Among the diarrheal specimens tested, ETEC strains were isolated from 14% (1996–1998) and 11% (2007–2012) of the patients of all age groups. During the six year study period, ETEC strains produced, 43% of LT/ST toxin, 27% of LT and 30% of ST toxin type. The isolation rates for ST-ETEC and LT/ST ETEC during 2007–2012 were significantly different from those seen for ETEC isolated between 1996–1998 (p<0.05) (Table 1). Among ST variants, STp was isolated in 5% of patients (Table 2). During the recent study period about 52% of ETEC was isolated from those over five years of age and 48% in younger age groups. The toxin profiles did not differ significantly in the different age groups studied (Table 3).

**Table 1:** Toxin profile of ETEC strains isolated from diarrheal patients in Bangladesh during two study periods.

	Relative distribution of ETEC with different toxin profile (%)		
Toxin types	1996–1998*	2007–2012	p value
LT	25	27	NS
ST	49	30	0.009
LT/ST	25	43	0.011

*: Qadri *et.al* 2000.

NS: Not significant.

**Table 2:** Relative distribution of STh and STp during the study period.

Year	STh	STp	STh/STp ratio
2007	62 (21)	8 (3)	62/8 (7.8)
2008	64 (20)	23 (7)	64/23 (2.8)
2009	68 (24)	16 (6)	68/16 (4.3)
2010	55 (27)	8(4)	55/8 (6.9)
2011	78 (33)	8(3)	78/8 (9.8)
2012	91 (29)	17 (5.4)	91/17 (5.4)
2007–2012	418	80	418/80 (5.2)

**Table 3:** Incidence of diarrhea caused by ETEC of different toxin phenotypes in different age groups among 2% of all diarreal patients seeking care at the icddr,b hospital in Bangladesh during 2007–2012.

Age	Total diarrhea N = 15152 (%)^b^	ETEC toxin phenotypes N (%)^a^			
		LT	ST	LT/ST	Total ETEC
0–5 months	1036 (6.8)	32 (3.0)	32 (3.0)	36 (3.4)	100 (9.6)
6–11 months	3259 (21.5)	113 (3.4)	89 (2.7)	120 (3.6)	322 (9.8)
12–17 months	1535 (10.1)	50 (3.2)	43 (2.8)	46 (2.9)	139 (9.05)
18–23 months	677 (4.5)	23 (3.3)	28 (4.1)	36 (5.3)	87 (12.8)
2–5 years	1113 (7.4)	38 (3.4)	60 (5.3)	52 (4.6)	150 (13.4)
>5 years	7520 (49.7)	186 (2.4)	246 (3.2)	427 (5.6)	859 (11.4)

a/N = Number of cases, % of all diarrheal cases.

b = % of cases in different age groups.

In patients with co- infections, rotavirus was the most common co pathogen with ETEC (17.2%), followed by *Vibrio cholerae* O1 (16.3%), *Campylobacter* spp (5.7%), *Shigella* spp (2.5%), and *Salmonella* spp (1.0%). Mixed infection together with ETEC and rotavirus occurred mostly in children below five years of age (14.6%) whereas mixtures of ETEC and *Vibrio cholerae* occurred mainly in patients above five years (12.0%).

A comparison was performed to determine if there is an association between the presence of toxin profile and the clinical severity of ETEC diarrhea (Table 4); from 2007 to 2009 we observed ETEC diarrhea of the same frequencies irrespective of toxin profile of the infecting strains. During 2010–2012, however, there was a significant difference (p = 0.046) in severity of ETEC diarrhea between strains producing LT only and those producing both LT/ST toxins. According to WHO, severe diarrhea is defined as episodes of diarrhea with fever and vomiting, requiring intravenous rehydration and needs hospitalization [17].

**Table 4:** Relationship between toxin profile and dehydration status of ETEC diarrheal patients during 2007–2012.

Dehydration Status^a^		Toxin profile no. (%)		
		LT	ST	LT/ST
Severe	2007–2009	60 (36.6)	47 (28.7)	57 (34.8)
	2010–2012	22 (17.2)	44 (34.4)	62 (48.4)
Some	2007–2009	82 (34.2)	75 (31.3)	83 (34.6)
	2010–2012	48 (26.8)	55 (30.7)	76 (42.5)

p value for chi-squared test: 0.046 (2010–2012); indicates LT vs LT/ST ETEC for severe cases.

a Degree of dehydration based on World Health Organization guideline.

### Colonization Factors

Among the ETEC positive isolates collected between 2007–2012, 49% (n = 812) expressed one or more of the 13 tested CFs as determined by dot blot immunoassay. The predominant CF types were CS5+CS6 (18%), CFA/I (14%), CS7 (12%), CS6 as well as CS17 (10%) and CS14 (9%) (Table 5). ETEC expressing CS5+CS6 (11%), CS7 (8%), and CS17 (3%) were isolated less frequently during the previous 1996 –1998 study period.

**Table 5:** Relative distribution of colonization factors (CFs) in ETEC isolates from diarrhea cases in Bangladesh according to year of isolation and association of specific CFs with toxin profile.

Percent of all CF positive strains distribution by years							Total no. of CFs
CF types	2007	2008	2009	2010	2011	2012	
	n = 144	n = 173	n = 115	n = 96	n = 123	n = 161	n = 812
	(%)	(%)	(%)	(%)	(%)	(%)	(%) of all CF positives
CFA/I±CS21	17.4	13.9	13.0	12.5	14.6	20.5	127 (15.6)
CS1+CS3±C21	9.7	4.0	6.1	7.3	10.6	9.9	64 (7.9)
CS2+CS3±CS21	5.5	8.0	7.0	7.3	5.7	3.1	49 (6.0)
CS4+CS6	6.9	6.9	2.6	3.1	4.9	5.6	45 (5.5)
CS5+CS6	16.0	19.1	20.9	25.0	18.7	13.7	149 (18.3)
CS6 only	8.3	8.1	14.8	9.4	8.1	9.9	78 (9.6)
CS6±CS8	4.8	1.7	5.2	1.0	1.6	0	20 (2.5)
CS7	13.9	23.7	10.4	12.5	6.5	5.0	101 (12.4)
CS12	2.1	0.6	1.7	0	1.6	1.9	11 (1.4)
CS14	8.3	6.4	6.1	5.2	14.6	14.9	77 (9.5)
CS17	6.3	6.4	8.7	14.6	12.2	14.3	82 (10.0)
CS21 only	0.7	0.6	1.7	2.1	0.8	1.2	9 (1.1)

Marked difference in CF profiles of ETEC isolates during 2007–2012 were observed particularly for strains expressing CS7, CS14 and CS17 (Table 5). About 51% (n = 845) of the CF negative strains identified between 2007–2012 were also tested for presence of genes for CS13, CS15, CS18, CS19, CS20 and CS22 antigens but none of them were positive for any of these colonization factors.

### ‘O’-Serogroups on ETEC Isolates

The most common serogroups on the CF positive ETEC isolates were O115 (16%), O114 (15%), O6 (13%), O25 (9%), O8 (7%) while 3% were non-typeable (NT). We found associations between the presence of CFs and ‘O’ serogroups of the isolates: CS5+CS6 (O115 and O126), CS7 (O114), CFA/I (O78 and O126), CS17 (O8 and O167) and CS1/CS2+CS3 (O6). Serogroup O1 was detected in the case of CS8 co-express with or without CS6 ETEC strains (Table 6).

**Table 6:** ‘O’-Serogroups on ETEC strains expressing different combinations of toxins and CFs during the study period (2007–2012).

Major Toxin Profiles	Colonization factors (CFs)	‘O’ Serogroup	
		Major	Minor
ST, LT/ST	CFA/I ± CS21 (61)	O78 (9), O126 (12), NT (15), OR (5), O? (10)	O128 (3), O8 (2), O114 (1), O15 (1), O6 (1), O55 (1), O44 (1)
ST, LT/ST	CS1+ CS3 ± CS21 (42)	O6 (34), NT (7)	O? (1)
ST, LT/ST	CS2+ CS3 ± CS21 (29)	O6 (26)	O115 (1), O2 (1), NT (1)
ST, LT/ST	CS4+ CS6 (24)	O25 (23)	NT (1)
ST, LT/ST	CS5+ CS6 (100)	O115 (87)	O126 (3), O167 (2), O169 (1), O20 (1), O27 (1), NT (2), OR (2), O? (1)
LT, ST, LT/ST	CS6 (46)	O159 (6), OR (7), NT (14),	O167 (3), O25 (3), O27 (2), O18 (2), O115 (1), O151 (1), O148 (2), O78 (1), O20 (1, OI69 (1), O? (2),
LT	CS6+ CS8 (14)	O25 (11)	O159 (1), O158 (1), O1 (1)
LT	CS7 (64)	O114 (60)	O115 (1), O86 (1), NT (1), OR (1)
LT	CS8 (2)	O1 (2)	-
LT, LT/ST	CS12 (7)	O18 (5)	NT (2)
LT/ST	CS14 (27)	NT (21)	O6 (2), O8 (1), O86a (1), O78 (1), O167 (1)
LT	CS17 (48)	O8 (36),	O167 (4), O6 (1), O153 (1), O20 (1), NT (3), OR (1), O? (1)
ST	CS21 (4)	O6 (3)	O128 (1)
LT, ST, LT/ST	Negative (130)	O8 (7), O114 (6), O20 (5), O153 (5), O25 (5), O15 (5), OR (5), O? (15), NT (59)	O18 (4), O27 (3), O1 (2), O86a (3), O159 (2), O78 (2), O6 (1), O128 R(1),

NT = Not Typeable.

OR = Rough strains.

O? = Polyvalent positive, mono valent negative.

## Discussion

Our present analysis shows that ETEC was isolated from diarrheal patients of different age groups (from less than 1 month to over 97 years of age). We observed that ETEC was a major cause of diarrhea not only in those less than five years of age but also in older children and adults. Earlier analyses including the surveillance carried out in the same site have shown that ETEC was more frequently isolated from children than from adults [4], [8], [18]. In this study, we observed that the relative proportion of ST- only ETEC had decreased significantly compared to that seen in a previous study in Bangladesh [8].

ETEC strains producing ST have previously been found to be more common in children with symptomatic infections where almost 80% were ST or LT/ST expressing strains [19] and also ST has been found to be prevalent in Egypt [18]. The isolation rate of LT/ST ETEC was higher in patients during the study period, which was also the most prevalent type of ETEC in Peru [20]. We also observed that colonization factor negative LT/STp producing ETEC strains were significantly higher in frequency than colonization factor expressing LT/STp ETEC strains. In case of STp- ETEC strains there was no difference in proportion between the CF-positive and CF-negative STp ETEC in our study.

Previous studies in Egypt and Guatemala have shown that there is almost as high frequency of STp compared to STh positive ETEC strains isolated from diarrheal stools of children [5]. However, in Bangladesh, we found STh producing ETEC to be much higher than the STp phenotype. One explanation may be the less prevalent consumption of porcine meat and products in the Bangladeshi settings.

During the study period between 2007–2012, colonization factors were identified almost in the same proportion of ETEC expressing LT, ST or both LT/ST toxins. However in earlier time periods, CF positive strains those were mainly observed among ST or LT/ST toxin types whereas LT only strains expressed a CF in low frequencies [8], [19]. A low prevalence of CFs on LT- ETEC strains has also been observed in Argentina and Egypt [8], [21], [22].

During an earlier analysis between 1996 –1998 in the icddr,b hospital, CS5+CS6 were the most common CFs; it was also common in the recent period except 2008 and 2012. It was interesting to see that the prevalence of the colonization factor CS7 on ETEC peaked during 2008 which was an epidemic caused during a flood in Dhaka. The CF types expressed by the LT only ETEC were mostly CS7 and CS17, while the LT/ST positive isolates expressed the prevalent CS5+CS6, CS14, CS1+CS3 or CFA/I.

ETEC isolates expressing CS7 and CS17 were previously found only in stools of diarrheal children aged less than 5 years [8]. Our present study shows that, from 2010 to 2012, both CS7 and CS17 as well as CFA/I are higher in isolation amongst younger children. However, CS5+CS6-expressing ETEC were more commonly prevalent in the older children and adults. In the previous study it was also found that some CS7-expressing isolates produced only ST, however during this later surveillance lasting for a six year period, no CS7 expressing ST-ETEC was identified. We found in our present study CS21 associated with the bacteria expressing coli surface antigens CS1/CS2+CS3 and CFA/I which was also observed earlier [8], [23].

ETEC can cause severe cholera like disease but also mild and moderate forms of illness. In the period between 2010–2012, there was a significant difference between severity of ETEC diarrhea and toxin profiles which was similar to our previous findings in 1996–1998 [8]. However, we found that in the study period from 2007 to 2009, there was no relation to the toxin type with the severity of diarrhea. In recent observation in Bolivia there was also no difference between severity of ETEC diarrhea and toxin profile on ETEC isolates [24].

The usefulness of ‘O’ serogroup antigens in ETEC vaccine formulation has been limited due to the large variation of ‘O’ serogroups, with over 80 serogroups identified among clinical ETEC isolates [25]. However, before the characterization of CFs, some ‘O’ serogroups such as O6, O8, O78, O128 and O153 have been shown to commonly associated with ETEC strains [26]. Similar association between ‘O’ groups and CF types were seen in most cases in the present study. Our results suggest that the shift observed in CF profile over time was also noted for ETEC strains in Bangladesh over the study period. Although there have been some shifts in CF antigenic types over time and it seems that the majority of ETEC still express the major CFs i.e. CS1-CS6 [2], [3] with occasional outbreaks of the closely related CS7, CS14 and CS17 all of which belong to the CFA/I group of antigens [27].

One of the limitations of our study was that we screened only the ETEC positive isolates for 13 of the 25 CFs that are known to exist and are common [2], using specific monoclonal antibodies. Six more CFs (CS13, CS15, CS18, CS19, CS20, CS22) were tested by using specific primers. However, neither monoclonal antibodies nor specific primers were available in our lab for detection of any remaining CFs that may have been present.

Vaccine formulation and development therefore should be based on the prevailing CF phenotypes of ETEC strains. We feel that the formulation of an effective cocktail for designing a multivalent ETEC vaccine should include CFs being isolated in high frequencies in recent studies in Bangladesh (e.g. CS7, CS17) and elsewhere [28].

## Supporting Information

Checklist S1 STROBE checklist.
(DOC)

## References

[1] Fleckenstein JM, Hardwidge PR, Munson GP, Rasko DA, Sommerfelt H, et al (2010) Molecular mechanisms of enterotoxigenic Escherichia coli infection. Microbes Infect 12: 89–98. 19883790 10.1016/j.micinf.2009.10.002 PMC10647112

[2] Gaastra W, Svennerholm AM (1996) Colonization factors of human enterotoxigenic Escherichia coli (ETEC). Trends Microbiol 4: 444–452. 8950814 10.1016/0966-842x(96)10068-8

[3] Isidean SD, Riddle MS, Savarino SJ, Porter CK (2011) A systematic review of ETEC epidemiology focusing on colonization factor and toxin expression. Vaccine 29: 6167–6178. 21723899 10.1016/j.vaccine.2011.06.084

[4] Qadri F, Svennerholm AM, Faruque AS, Sack RB (2005) Enterotoxigenic Escherichia coli in developing countries: epidemiology, microbiology, clinical features, treatment, and prevention. Clin Microbiol Rev 18: 465–483. 16020685 10.1128/CMR.18.3.465-483.2005 PMC1195967

[5] Bolin I, Wiklund G, Qadri F, Torres O, Bourgeois AL, et al (2006) Enterotoxigenic Escherichia coli with STh and STp genotypes is associated with diarrhea both in children in areas of endemicity and in travelers. J Clin Microbiol 44: 3872–3877. 16943355 10.1128/JCM.00790-06 PMC1698338

[6] Grewal HM, Valvatne H, Bhan MK, van Dijk L, Gaastra W, et al (1997) A new putative fimbrial colonization factor, CS19, of human enterotoxigenic Escherichia coli. Infect Immun 65: 507–513. 9009305 10.1128/iai.65.2.507-513.1997 PMC176088

[7] Pichel M, Binsztein N, Viboud G (2000) CS22, a novel human enterotoxigenic Escherichia coli adhesin, is related to CS15. Infect Immun 68: 3280–3285. 10816474 10.1128/iai.68.6.3280-3285.2000 PMC97580

[8] Qadri F, Das SK, Faruque AS, Fuchs GJ, Albert MJ, et al (2000a) Prevalence of toxin types and colonization factors in enterotoxigenic Escherichia coli isolated during a 2-year period from diarrheal patients in Bangladesh. J Clin Microbiol 38: 27–31. 10618058 10.1128/jcm.38.1.27-31.2000 PMC86010

[9] WHO (1987) Programme for control of diarrheal diseases. In manual for laboratory investigation of Acute Enteric Infections. Document CDD/93.3; Rev 1. Geneva, Switzerland. 9–20.

[10] Rodas C, Iniguez V, Qadri F, Wiklund G, Svennerholm AM, et al (2009) Development of multiplex PCR assays for detection of enterotoxigenic Escherichia coli colonization factors and toxins. J Clin Microbiol 47: 1218–1220. 19244463 10.1128/JCM.00316-09 PMC2668350

[11] Sjoling A, Wiklund G, Savarino SJ, Cohen DI, Svennerholm AM (2007) Comparative analyses of phenotypic and genotypic methods for detection of enterotoxigenic Escherichia coli toxins and colonization factors. J Clin Microbiol 45: 3295–3301. 17687011 10.1128/JCM.00471-07 PMC2045327

[12] Steinsland H, Valentiner-Branth P, Grewal HM, Gaastra W, Molbak KK, et al (2003) Development and evaluation of genotypic assays for the detection and characterization of enterotoxigenic Escherichia coli. Diagn Microbiol Infect Dis 45: 97–105. 12614980 10.1016/s0732-8893(02)00504-7

[13] Svennerholm AM, Wikstrom M, Lindblad M, Holmgren J (1986) Monoclonal antibodies against Escherichia coli heat-stable toxin (STa) and their use in a diagnostic ST ganglioside GM1-enzyme-linked immunosorbent assay. J Clin Microbiol 24: 585–590. 2429984 10.1128/jcm.24.4.585-590.1986 PMC268976

[14] Svennerholm AM, Wiklund G (1983) Rapid GM1-enzyme-linked immunosorbent assay with visual reading for identification of Escherichia coli heat-labile enterotoxin. J Clin Microbiol 17: 596–600. 6343419 10.1128/jcm.17.4.596-600.1983 PMC272699

[15] Qadri F, Giron JA, Helander A, Begum YA, Asaduzzaman M, et al (2000b) Human antibody response to longus type IV pilus and study of its prevalence among enterotoxigenic Escherichia coli in Bangladesh by using monoclonal antibodies. J Infect Dis 181: 2071–2074. 10837196 10.1086/315507

[16] Begum YA, Talukder KA, Nair GB, Khan SI, Svennerholm AM, et al (2007) Comparison of enterotoxigenic Escherichia coli isolated from surface water and diarrhoeal stool samples in Bangladesh. Can J Microbiol 53: 19–26. 17496946 10.1139/w06-098

[17] WHO (1990) Treatment of diarrhoea: A manual for physicians anal other senior health workers. Geneva, Swiitzerland: World Health Organization.

[18] Shaheen HI, Khalil SB, Rao MR, Abu Elyazeed R, Wierzba TF, et al (2004) Phenotypic profiles of enterotoxigenic Escherichia coli associated with early childhood diarrhea in rural Egypt. J Clin Microbiol 42: 5588–5595. 15583286 10.1128/JCM.42.12.5588-5595.2004 PMC535259

[19] Qadri F, Saha A, Ahmed T, Al Tarique A, Begum YA, et al (2007) Disease burden due to enterotoxigenic Escherichia coli in the first 2 years of life in an urban community in Bangladesh. Infect Immun 75: 3961–3968. 17548483 10.1128/IAI.00459-07 PMC1951985

[20] Rivera FP, Ochoa TJ, Maves RC, Bernal M, Medina AM, et al (2010) Genotypic and phenotypic characterization of enterotoxigenic Escherichia coli strains isolated from Peruvian children. J Clin Microbiol 48: 3198–3203. 20631096 10.1128/JCM.00644-10 PMC2937683

[21] Rao MR, Abu-Elyazeed R, Savarino SJ, Naficy AB, Wierzba TF, et al (2003) High disease burden of diarrhea due to enterotoxigenic Escherichia coli among rural Egyptian infants and young children. J Clin Microbiol 41: 4862–4864. 14532244 10.1128/JCM.41.10.4862-4864.2003 PMC254374

[22] Viboud GI, Jouve MJ, Binsztein N, Vergara M, Rivas M, et al (1999) Prospective cohort study of enterotoxigenic Escherichia coli infections in Argentinean children. J Clin Microbiol 37: 2829–2833. 10449460 10.1128/jcm.37.9.2829-2833.1999 PMC85388

[23] Harris AM, Chowdhury F, Begum YA, Khan AI, Faruque AS, et al (2008) Shifting prevalence of major diarrheal pathogens in patients seeking hospital care during floods in 1998, 2004, and 2007 in Dhaka, Bangladesh. Am J Trop Med Hyg 79: 708–714. 18981509 PMC2749297

[24] Gonzales L, Sanchez S, Zambrana S, Iniguez V, Wiklund G, et al (2013) Molecular characterization of enterotoxigenic Escherichia coli isolates recovered from children with diarrhea during a 4-year period (2007 to 2010) in Bolivia. J Clin Microbiol 51: 1219–1225. 23390275 10.1128/JCM.02971-12 PMC3666779

[25] Wolf MK (1997) Occurrence, distribution, and associations of O and H serogroups, colonization factor antigens, and toxins of enterotoxigenic Escherichia coli. Clin Microbiol Rev 10: 569–584. 9336662 10.1128/cmr.10.4.569 PMC172934

[26] Merson MH, Sack RB, Islam S, Saklayen G, Huda N, et al (1980) Disease due to enterotoxigenic Escherichia coli in Bangladeshi adults: clinical aspects and a controlled trial of tetracycline. J Infect Dis 141: 702–711. 6248600 10.1093/infdis/141.6.702

[27] Anantha RP, McVeigh AL, Lee LH, Agnew MK, Cassels FJ, et al (2004) Evolutionary and functional relationships of colonization factor antigen i and other class 5 adhesive fimbriae of enterotoxigenic Escherichia coli. Infect Immun 72: 7190–7201. 15557644 10.1128/IAI.72.12.7190-7201.2004 PMC529125

[28] Rodas C, Mamani R, Blanco J, Blanco JE, Wiklund G, et al (2011) Enterotoxins, colonization factors, serotypes and antimicrobial resistance of enterotoxigenic Escherichia coli (ETEC) strains isolated from hospitalized children with diarrhea in Bolivia. Braz J Infect Dis 15: 132–137. 21503399 10.1016/s1413-8670(11)70158-1

