# Supplementary material for: Shift in Phenotypic Characteristics of Enterotoxigenic Escherichia coli (ETEC) Isolated from Diarrheal Patients in Bangladesh
Source: PLoS Negl Trop Dis. 2014 Jul 17;8(7):e3031. doi: 10.1371/journal.pntd.0003031 (PMC4102457; doi:10.1371/journal.pntd.0003031)
Supplement: Checklist S1 — STROBE checklist. (DOC) [file pntd.0003031.s001.doc]

**Checklist S1: STROBE Checklist for: PNTD-D-14-00177R1: “Shift in Phenotypic Characteristics of Enterotoxigenic *Escherichia coli* (ETEC) Isolated from Diarrheal Patients in Bangladesh”**

|  | Item No | Recommendation and Answer |
| --- | --- | --- |
| **Title and abstract** | 1 | (*a*) Indicate the study’s design with a commonly used term in the title or the abstract  **ANSWER: Title:“Shift in Phenotypic Characteristics of Enterotoxigenic *Escherichia coli* (ETEC) Isolated from Diarrheal Patients in Bangladesh” (Page 01)** |
|  |  | (*b*) Provide in the abstract an informative and balanced summary of what was done and what was found  **ANSWER: Done in the Abstract section. (Page 02)** |
| Introduction | | |
| Background/rationale | 2 | Explain the scientific background and rationale for the investigation being reported  **ANSWER: Page 03, line 77-89** |
| Objectives | 3 | State specific objectives, including any prespecified hypotheses  **ANSWER: Page 03-04, line 89-92 (No prespecified hypothesis)** |
| Methods | | |
| Study design | 4 | Present key elements of study design early in the paper  **ANSWER: The study design is described in the Abstract (Page no. 02) as well as in the text Page 03-04, line 89-99** |
| Setting | 5 | Describe the setting, locations, and relevant dates, including periods of recruitment, exposure, follow-up, and data collection  **ANSWER:** **For setting, locations, and relevant dates: Page no. 04, line 96-97. Details on data collection: Page no. 04, line 104-114.** |
| Participants | 6 | (*a*) Give the eligibility criteria, and the sources and methods of selection of participants  **ANSWER: Eligibility criteria: Page no. 04, line 97-99 and selection of participants: Page no. 04, line 117-118** |
| Variables | 7 | Clearly define all outcomes, exposures, predictors, potential confounders, and effect modifiers. Give diagnostic criteria, if applicable  **ANSWER: Some outcomes such as degree of dehydration in diarrheal patients were assessed according to WHO guidelines. Page no. 04, line 97-98. Exposure to ETEC toxin and colonization factors: Page no. 06, line 150-159** |
| Data sources/ measurement | 8* | For each variable of interest, give sources of data and details of methods of assessment (measurement). Describe comparability of assessment methods if there is more than one group  **ANSWER: The data source for each variable of interest was obtained from two different time periods: during 1996-1998 and 2007-2012. Methods for detection of ‘ETEC toxin types’ and ‘colonization factors’ include PCR: (Page no. 05, line 120-145) and phenotypic methods: (Page no. 06, line 150-159). We have incorporated the results of the two methods (PCR and ELISA) employed for toxin type detection. For ‘O’ serogrouping: Page no. 06, line 162-164** |
| Bias | 9 | Describe any efforts to address potential sources of bias  **ANSWER: We enrolled every 50^th^ diarrheal patient and there was no bias in the study. Page no. 04, line 117-118** |
| Study size | 10 | Explain how the study size was arrived at  **ANSWER: During 2007-2012, we enrolled every 50^th^ diarrheal patients attending icddr,b hospital. Page no. 04, line 117-118 and Page no. 06, line 174-175** |
| Quantitative variables | 11 | Explain how quantitative variables were handled in the analyses. If applicable, describe which groupings were chosen and why  **ANSWER: The diarrheal patients were subdivided into two major groups: a) less than 5 years age and b) 5 years and older. Page no. 07, line 182-184** |
| Statistical methods | 12 | (*a*) Describe all statistical methods, including those used to control for confounding  **ANSWER: Page no. 06, line 167-169** |
|  |  | (*b*) Describe any methods used to examine subgroups and interactions |
|  |  | (*c*) Explain how missing data were addressed  **ANSWER: Missing data were very minor i.e. <1% and we excluded it from the analysis. Page no. 06, line 169-170** |
|  |  | (*d*) If applicable, describe analytical methods taking account of sampling strategy |
|  |  | (*e*) Describe any sensitivity analyses |
| Results | | |
| Participants | 13* | (a) Report numbers of individuals at each stage of study—eg numbers potentially eligible, examined for eligibility, confirmed eligible, included in the study, completing follow-up, and analysed  **ANSWER: Page no. 06, line 174-176** |
|  |  | (b) Give reasons for non-participation at each stage |
|  |  | (c) Consider use of a flow diagram |
| Descriptive data | 14* | (a) Give characteristics of study participants (eg demographic, clinical, social) and information on exposures and potential confounders  **ANSWER: Demographic: page no. 07, line 182-190 and clinical: Page no. 07, line 192-198** |
|  |  | (b) Indicate number of participants with missing data for each variable of interest  **ANSWER: Missing data were very minor i.e. <1% and we excluded it from the analysis. Page no. 06, line 169-170** |
| Outcome data | 15* | Report numbers of outcome events or summary measures  **ANSWER: Our study mainly compared descriptive data. Comparison of toxin types: (Table 1a.) and for colonization factors: Page 7-8, line 201-208 as well as (Table 4)** |
| Main results | 16 | (*a*) Give unadjusted estimates and, if applicable, confounder-adjusted estimates and their precision (eg, 95% confidence interval). Make clear which confounders were adjusted for and why they were included  **ANSWER: We only used unadjusted estimates in this study** |
|  |  | (*b*) Report category boundaries when continuous variables were categorized  **ANSWER: Category boundaries for age of diarrheal patients and ETEC toxin types are mentioned in Table 2** |
|  |  | (*c*) If relevant, consider translating estimates of relative risk into absolute risk for a meaningful time period  **ANSWER: Not relevant for our study** |
| Other analyses | 17 | Report other analyses done—eg analyses of subgroups and interactions, and sensitivity analyses  **ANSWER: Some subgroup analyses are reported throughout the results and all tables and are explained precisely.** |
| Discussion | | |
| Key results | 18 | Summarise key results with reference to study objectives  **ANSWER:** **Page no. 08, line 221-227** |
| Limitations | 19 | Discuss limitations of the study, taking into account sources of potential bias or imprecision. Discuss both direction and magnitude of any potential bias  **ANSWER: Limitation of the study: Page no. 10, line 283-287** |
| Interpretation | 20 | Give a cautious overall interpretation of results considering objectives, limitations, multiplicity of analyses, results from similar studies, and other relevant evidence  **ANSWER: Done throughout the Discussion section** **(Page no. 8-10, line 221-292)** |
| Generalisability | 21 | Discuss the generalisability (external validity) of the study results  **ANSWER: Done throughout the Discussion section (Page no. 8-10, line 229-292)** |
| Other information | | |
| Funding | 22 | Give the source of funding and the role of the funders for the present study and, if applicable, for the original study on which the present article is based  **ANSWER: Source of funding was listed during the submission**  **process and as far as we know will be mentioned on the side. To avoid reiteration we did not mention that in the text. The funders had no role in study design, data collection, analysis and publication. There are no conflicts of interest.** |

*Give information separately for exposed and unexposed groups.

**Note:** An Explanation and Elaboration article discusses each checklist item and gives methodological background and published examples of transparent reporting. The STROBE checklist is best used in conjunction with this article (freely available on the Web sites of PLoS Medicine at http://www.plosmedicine.org/, Annals of Internal Medicine at http://www.annals.org/, and Epidemiology at http://www.epidem.com/). Information on the STROBE Initiative is available at www.strobe-statement.org.
